# The Impact of Radiofrequency Ablation on Survival Outcomes and Stent Patency in Patients with Unresectable Cholangiocarcinoma: A Systematic Review and Meta-Analysis of Randomized Controlled Trials

**DOI:** 10.3390/cancers16071372

**Published:** 2024-03-30

**Authors:** Daniele Balducci, Michele Montori, Francesco Martini, Marco Valvano, Federico De Blasio, Maria Eva Argenziano, Giuseppe Tarantino, Antonio Benedetti, Emanuele Bendia, Marco Marzioni, Luca Maroni

**Affiliations:** 1 Clinic of Gastroenterology, Hepatology, and Emergency Digestive Endoscopy, Università Politecnica delle Marche, 60126 Ancona, Italy; 2 Gastroenterology Unit, Division of Gastroenterology, Hepatology, and Nutrition, Department of Life, Health and Environmental Sciences, University of L’Aquila, 67100 L’Aquila, Italy; 3 Division of Gastroenterology, Galliera Hospital, 16128 Genoa, Italy; 4 Division of Digestive Diseases, Digestive Endoscopy and Inflammatory Bowel Diseases, A.O.U. “Ospedali Riuniti”, 60126 Ancona, Italy

**Keywords:** radiofrequency ablation, cholangiocarcinoma, stenting, survival, meta-analysis

## Abstract

**Simple Summary:**

This study investigated the efficacy of combining radiofrequency ablation (RFA) with stenting versus stenting alone in treating inoperable cholangiocarcinoma. Through a systematic review and meta-analysis of randomized controlled trials, it assessed the impact of the treatment on overall survival and stent patency. Although the results show no significant difference in overall survival between the groups, there was a trend toward improved survival in the subgroup treated with RFA plus plastic stents. Stent patency was significantly better in the RFA group. Adverse events were not different between the groups. These findings suggest that RFA may offer benefits in palliative care for these patients, warranting further research to explore the potential cumulative effects of RFA.

**Abstract:**

Endoluminal biliary radiofrequency ablation (RFA) has been proposed as a palliative treatment for patients with malignant biliary obstruction (MBO) in order to improve stent patency and survival. However, the existing data on patients with inoperable extrahepatic cholangiocarcinoma (eCCA) are conflicting. We performed a meta-analysis of randomized trials comparing RFA plus stenting versus stenting alone in patients with inoperable eCCA. We searched for trials published in the PubMed/MEDLINE, Scopus, and Cochrane databases up to November 2023. Data extraction was conducted from published studies, and a quality assessment was carried out in accordance with the guidelines recommended by the Cochrane Collaboration. Hazard ratios (HRs) with 95% CI were estimated from the trials. The primary endpoints of interest were overall survival and stent patency. Out of 275 results, 5 randomized trials and 370 patients were included. While overall survival was not different between the groups (HR 0.62; 95% CI 0.36–1.07; *p* = 0.09; I^2^ = 80%;), the subgroup analysis of studies employing plastic stents showed a trend toward better survival in the RFA-treated group (HR 0.42; 95% CI 0.22–0.80; *p* = 0.009; I^2^ = 72%). Stent patency was improved in patients receiving RFA (HR 0.64; 95% CI 0.45–0.90; *p* = 0.01; I^2^ = 23%). Adverse events were not different between the groups (OR 1.21; 95% CI 0.69–2.12; *p* = 0.50; I^2^ = 0%). Despite the promising results, high heterogeneity and potential biases in the included studies suggest the need for further high-quality randomized trials to explore the potential cumulative effects of RFA on CCA treatment outcomes.

## 1. Introduction

Malignant biliary obstruction (MBO) emerging from extrahepatic cholangiocarcinoma (eCCA) poses a significant challenge in clinical oncology. As most patients are deemed inoperable upon diagnosis, a multidisciplinary approach is critical to assess treatment options, and particular focus is often placed on achieving optimal biliary drainage [1].

As such, endoscopic drainage stands out as a pivotal palliative procedure, particularly with the adoption of self-expandable metal stents (SEMSs), which have been shown to be superior to plastic stents in terms of patency, adverse event rates, cost-effectiveness, and mortality [2,3]. However, recurrent biliary obstruction, mainly caused by sludge accumulation or tumor ingrowth, poses a significant hurdle, emphasizing the demand for interventions that prolong stent patency and, consequently, reduce healthcare costs and enhance the patient’s quality of life [4]. With the extended life expectancy associated with novel anticancer therapies, newer ablation techniques such as radiofrequency ablation (RFA), Photodynamic Therapy, and Microwave Ablation have been implemented, aiming to extend stent patency and possibly survival [5,6].

Radiofrequency ablation (RFA) has been deployed as a localized therapeutic approach for several types of conditions, including heart diseases [7] and malignant neoplasms [8]. Tumor irradiation as a local ablative therapy, despite the relative radio-resistance of cholangiocarcinoma, represents a therapeutic option, and certain studies have demonstrated an increased survival rate compared to control groups, showcasing its potential efficacy [9]. RFA occurs through the generation of thermal damage caused by a high-frequency alternating current discharged from an electrode into tissue: by inducing coagulation necrosis in the targeted area, RFA offers a promising approach to achieving the local control of tumor growth [10,11,12]. As such, the implementation of endobiliary RFA has gained traction as an adjunctive tool in palliative treatment for malignant biliary obstruction. The initial cannulation of the bile duct can be conducted through an endoscopic or percutaneous approach and allows the doctor to ascertain the stricture’s length, diameter, and precise location. Subsequently, the radiofrequency (RF) catheter is maneuvered over the guidewire, following the strategic placement of radiopaque electrodes at the stricture. The determination of the stricture’s length is pivotal in establishing the appropriate electrode length for the RF catheter. The procedure involves the execution of overlapping RFA, transitioning from the distal to the proximal margin of the stricture, thereby enabling the complete obliteration of malignant strictures through serial overlapping ablations. The utilization of the RFA probe ensues, calibrated with predetermined energy, temperature, and duration settings. Upon the conclusion of ablation therapy, residual coagulated tissue debris may be excised via balloon sweeps, and a cholangiogram is employed for the detection of biliary complications, such as perforations. Since segmental biliary strictures can arise within four weeks post-endobiliary RFA due to fibrotic changes, these procedures are usually followed by biliary stent placement and are typically performed at the time of the first stent placement or its subsequent replacement [13,14,15]. This mechanism has prompted extensive research to explore its potential not only to enhance survival but also to extend the patency of biliary stents.

Existing data on the relationship between RFA, survival, and stent patency are inconsistent, as there are only a few prospective studies published on their use in patients with eCCA, and they report conflicting results [16,17,18,19,20].

While previous meta-analyses on this subject have indicated a favorable effect on survival outcomes and stent patency, they predominantly incorporated retrospective studies and a minimal number of randomized controlled trials (RCTs). Furthermore, these analyses broadly encompassed all causes of MBO, such as pancreatic cancer, carcinoma of the papilla, and metastasis, without discriminating among the various etiologies [21,22,23,24,25]. Hence, the aim of this study was to conduct a structured systematic review and meta-analysis of only RCTs to assess the impact of combining RFA with biliary stent placement on both overall survival and stent patency in patients with inoperable eCCA.

## 2. Materials and Methods

### 2.1. Search Strategy and Selection Criteria

The systematic review and meta-analysis were performed in accordance with recommendations from the Cochrane Collaboration and the Preferred Reporting Items for Systematic Reviews and Meta-Analysis (PRISMA) statement guideline (Supplementary Table S1) [26]. We used a predetermined protocol (https://doi.org/10.17605/OSF.IO/9CRG3, accessed on 27 February 2024). We systematically searched Scopus, the Cochrane Central Register of Controlled Trials, and PubMed from inception to November 2023 for studies published in English with the following medical terms and their variations: “cholangiocarcinoma”, “endoluminal radiofrequency”, and “stenting”. In addition, the references of the included studies and systematic reviews were manually evaluated for additional studies. A complete electronic search strategy is reported in Appendix A.

Two authors (D.B. and M.M.) individually reviewed the abstracts and full texts for eligibility. Conflicts were resolved by referring to the original articles. The selection was made according to the following criteria: (1) randomized controlled trials (RCTs); (2) comparing RFA plus stenting with stenting alone; (3) in patients with inoperable cholangiocarcinoma; and (4) reporting at least one of the clinical outcomes of interest (overall survival or stent patency). We excluded studies (1) with overlapping patient populations or (2) without a control group with stenting alone. RCTs of RFA plus stenting in patients with malignant biliary obstruction were included only if they reported dedicated outcomes in the cholangiocarcinoma population.

We extracted data for (1) overall survival; (2) stent patency; and (3) adverse events. In the case of lacking/missing data, the authors of the eligible studies were contacted to obtain the missing information. Overall survival and stent patency were compared using pooled hazard ratios (HRs) to preserve time-to-event data from individual studies, whereas adverse events were compared using pooled Odds Ratios (ORs).

### 2.2. Data Extraction and Statistical Analysis

Baseline characteristics and outcomes were independently extracted by two researchers (D.B. and M.M.) based on predetermined criteria for searching, data extraction, and quality evaluation, as reported in Table 1. Any disagreements that arose were resolved through consensus among three researchers (D.B., M.M., and F.M.). Treatment effects for overall survival and stent patency were compared using pooled HRs with 95% confidence intervals in order to preserve time-to-event data, while adverse events were compared with pooled ORs with 95% confidence intervals. In this meta-analysis, the estimation of HRs for comparing outcomes across studies was conducted using two different statistical approaches, depending on the granularity and format of the data available from the included studies. For studies that provided detailed individual patient data, including the time to event or censoring, we employed the Cox proportional hazards model. This approach allowed for a more nuanced analysis, taking into account the timing of each event and providing a robust estimate of the HR. In contrast, for studies where only aggregated survival data were available, such as median survival times and their corresponding 95% confidence intervals, we used an estimation method as proposed by Parmar et al. [27]. This method, while more approximate, enabled the calculation of HRs from summary statistics, ensuring that all available study data could contribute to the meta-analysis, despite variations in data reporting. The use of these complementary approaches allowed for a comprehensive and inclusive analysis of survival outcomes, maximizing the utility of diverse data sources while acknowledging the limitations inherent in each method. The assessment of heterogeneity was conducted using the Cochran Q test and I^2^ statistics, with *p* values below 0.10 and I^2^ values over 25% indicating significant heterogeneity. We used the DerSimonian and Laird random-effects model for all endpoints, and *p* < 0.05 was considered statistically significant. Review Manager 5.3 (Cochrane Center, The Cochrane Collaboration, Denmark) and R Statistical Software (version 4.3.2; R Foundation for Statistical Computing, Vienna, Austria) were used for statistical analysis.

### 2.3. Risk-of-Bias Assessment and Sensitivity Analysis

The risk of bias was independently assessed by two reviewers (D.B. and M.M.) using the Cochrane Collaboration’s tool version 2 (RoB 2) [28].

Publication bias was planned to be investigated using funnel plots for outcomes whose data are available from ten or more studies. Egger’s regression test was also planned for primary outcome analysis in order to assess potential publication bias [29].

A sensitivity analysis was planned to be conducted, excluding studies with a high risk of bias assessed by the RoB 2.

The quality of evidence for each outcome was planned to be assessed by GRADE, also providing a summary of findings.

## 3. Results

### 3.1. Study Selection and Baseline Characteristics

**Figure 1:**
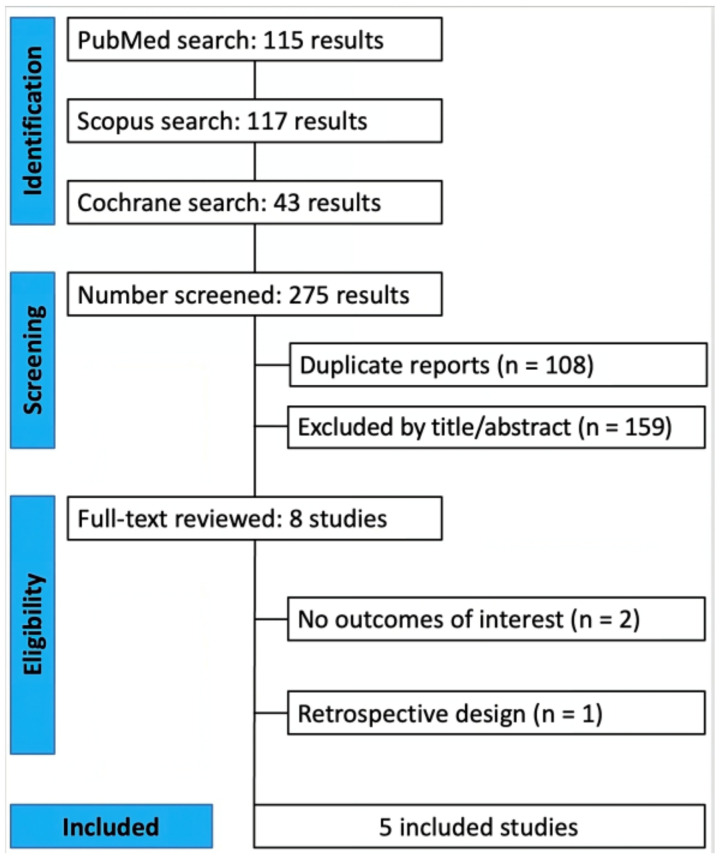
PRISMA flow diagram of study screening and selection.

As detailed in Figure 1, the initial search yielded 275 results. After the removal of duplicate records and ineligible studies, eight remained and were fully reviewed based on the inclusion criteria. Of these, a total of five studies were included, comprising 370 total patients [16,17,18,19,20]. The study characteristics are reported in Table 1. Two studies used self-expandable metal stents (SEMSs) [19,20], whereas three studies used plastic stents and allowed repeated treatment with RFA [16,17,18]. In particular, Yang et al. [17] and Gao et al. [18] utilized 8.5 Fr plastic biliary stents. Kang et al. [16] employed 7 or 8.5 Fr plastic biliary stents. Andrasina et al. [20] utilized uncovered SEMSs. Jarosova et al. [19] used uncovered SEMSs in patients with hilar CCA and partially covered SEMSs in patients with distal strictures; plastic stents were used in 11% of cases where metal stent insertion was technically impractical. Across all included studies, the stent length was selected based on the lengths of the strictures.

Only one study used a percutaneous technique [20]. The HR for the overall survival was estimable for all the included studies, while the HR for the stent patency was estimable for four studies [16,17,19,20].

Four studies [16,17,18,19] compared adverse events between the groups.

### 3.2. Pooled Analysis of All Studies

**Figure 2:**
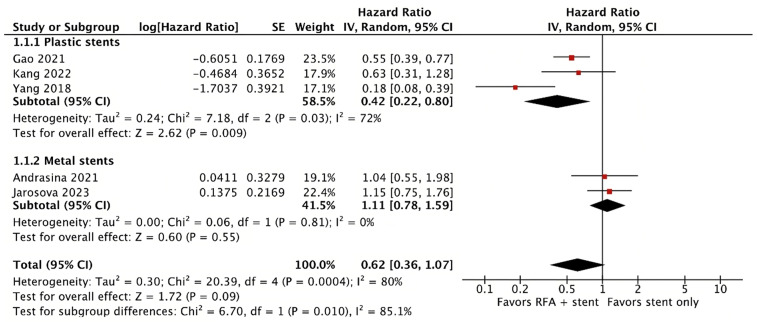
Pooled hazard ratios (HRs) for overall survival [16,17,18,19,20].

**Figure 3:**
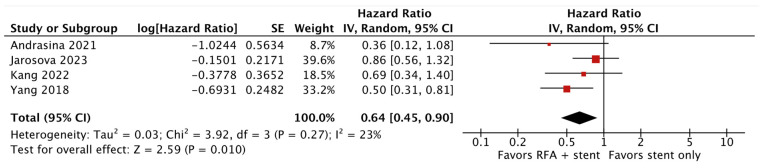
Pooled hazard ratios (HRs) for stent patency [16,17,19,20].

**Figure 4:**
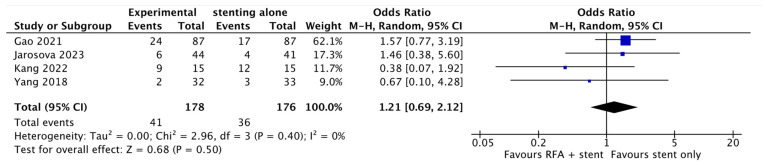
Pooled analysis of adverse events [16,17,18,19].

The analysis included 370 patients, with a balanced proportion between male and female patients (male sex 52%). The tumor location was distributed as follows: distal 42.8%, Bismuth I–II 23.7%, Bismuth III 19.7%, and Bismuth IV 13.8%. The result of the primary outcome reported in this meta-analysis showed no statistical difference between groups in terms of overall survival (HR 0.62; 95% CI 0.36–1.07; *p* = 0.09; I^2^ = 80%; Figure 2). However, despite the high heterogeneity, there was a trend toward better overall survival in patients receiving RFA plus plastic stents (HR 0.42; 95% CI 0.22–0.80; *p* = 0.009; I^2^ = 72%; Figure 2). On the other hand, stent patency was improved in the group receiving RFA plus stenting (HR 0.64; 95% CI 0.45–0.90; *p* = 0.01; I^2^ = 23%; Figure 3). A pooled analysis of adverse events showed no statistical difference between the groups (OR 1.21; 95% CI 0.69–2.12; *p* = 0.50; I^2^ = 0%; Figure 4).

### 3.3. Quality Assessment

**Figure 5:**
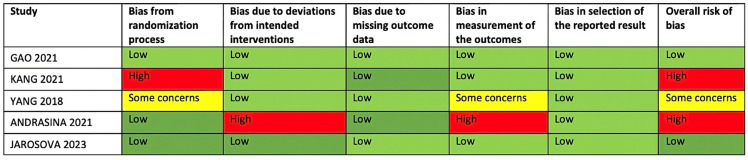
Risk-of-bias summary for randomized studies (RoB 2) [16,17,18,19,20].

The Risk of Bias 2 (RoB 2) tool was used to perform the quality assessment [28]. As shown in Figure 5, two studies were considered at high risk of bias (mainly due to the risk of bias in the randomization process and a bias in the measurement of the outcomes) [16,20]. One study was considered to have some concerns [17], and two studies were considered at low risk of bias [18,19].

The quality of evidence for each outcome was also assessed by GRADE, as shown in Table 2.

### 3.4. Sensitivity Analyses and Investigation of Publication Bias

Publication bias could not be adequately assessed using the funnel plot, nor could Egger’s regression test, due to the small number of selected studies for both primary and secondary outcomes.

A sensitivity analysis was performed, excluding the studies with a high risk of bias [16,20]. The result was comparable for overall survival (HR 0.51; 95% CI 0.22–1.19; *p* = 0.12; I^2^ = 89%), but no statistical difference was found in terms of stent patency (HR 0.66; 95% CI 0.39–1.13; *p* = 0.13; I^2^ = 63%)

**Figure 6:**
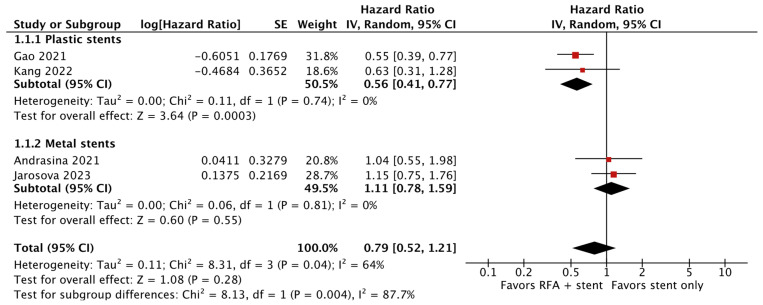
Post hoc analysis without Yang et al. [16,18,19,20].

A post hoc analysis was performed with the exclusion of Yang et al. [17], the only RCT without patients with complex strictures. This analysis revealed a low heterogeneity for both the subgroups of studies employing plastic stents (HR 0.56; 95% CI 0.41–0.77; *p* < 0.05; I^2^ = 0%; Figure 6) and metal stents (HR 1.11; 95% CI 0.78–1.59; *p* = 0.81; I^2^ = 0%; Figure 6) and a lower overall heterogeneity (HR 0.79; 95% CI 0.52–1.21; *p* = 0.28; I^2^ = 64%; Figure 6).

## 4. Discussion

In this systematic review and meta-analysis, which encompasses five studies and 370 patients, we explored the efficacy of RFA with stenting versus stenting alone in enhancing OS and stent patency among patients with unresectable cholangiocarcinoma receiving palliative treatment. Our primary findings reveal a notable trend toward improved OS for patients treated with RFA in conjunction with plastic stents, along with improved stent patency in the RFA-treated group. However, it is paramount to acknowledge that the OS was influenced by considerable heterogeneity across the pooled data.

CCA is a predominant cause of MBO, with most patients receiving a diagnosis at an advanced stage. This necessitates palliative treatments, such as chemotherapy or radiotherapy, which, unfortunately, offer limited efficacy. For that reason, novel palliative local treatments are under investigation in order to improve survival. RFA is a procedure that induces thermal damage through the application of a high-frequency alternating current emitted from an electrode into biological tissues. This process leads to coagulative necrosis and cellular death when the temperature threshold surpasses 50 °C. With the recent development of catheter-based RFA, it is now possible to apply RFA directly into bile ducts with both endoscopic and percutaneous techniques, with potential improvement in survival and stent patency. In fact, it has been hypothesized that the ablative procedure triggers a systemic immune response. This response is potentially enhanced by immune-modulating agents, leading to better clinical outcomes [30,31].

To explore the potential clinical benefits, some retrospective studies and RCTs have been conducted but show conflicting results [16,17,18,19,20,32,33,34,35]. Although previous meta-analyses published on this topic reported a positive impact of RFA on survival outcomes, they included mostly retrospective studies and very few RCTs, and most of them took into consideration all the etiologies of MBO, including pancreatic cancer, carcinoma of the papilla, and metastasis [21,22,23,24,25]. A recent meta-analysis of RCTs by de Oliveira Veras et al. [36] showed improved OS (mean difference 83.14 days (95% CI 29.52–136.77; I^2^ = 97%; *p* < 0.01) and improved stent patency (mean difference 76.73 days; 95% CI 50.11–103.34; I^2^ = 67%; *p* < 0.01). However, they (1) included fewer RCTs in the subgroup analysis of patients with cholangiocarcinoma and (2) did not take into account the differences in OS and stent patency using HRs in order to preserve time-to-event data.

Our analysis showed that, globally, RFA plus stenting is not superior to stenting alone for increasing OS. However, the subgroup analysis of patients receiving a plastic stent with RFA demonstrated a trend toward improved OS compared to stenting alone. This result could be attributed to the fact that the studies included in this subgroup permitted repeated RFA treatments, whereas such flexibility was not available in the studies utilizing metal stents. This observation underscores the potential for a cumulative therapeutic effect of RFA, warranting further investigation through prospective randomized studies comparing RFA with either stent type. Concerning stent patency, our analysis confirmed an improvement in the RFA-treated group. No statistical difference was found in terms of adverse events between the groups, suggesting that the RFA treatment does not increase the risks for the patients compared to the stenting procedure alone.

This study has limitations. First, only a few RCTs exploring the effect of RFA in patients with extrahepatic cholangiocarcinoma are available. For that reason, only five RCTs were included in our meta-analysis, and the pooled analysis for stent patency was performed using only four studies. Moreover, the pooled analysis of OS was affected by high heterogeneity. This can be explained by a different patient selection in terms of tumor localization and the utilization of different stent types across the included studies. Contrary to the other studies, Yang et al. [17] recruited patients with distal or Bismuth I-II stenosis only. They reported a mean survival in the RFA-treated group that was 5 months longer than survival in the untreated group. The exclusion of patients with complex strictures, which are the most common in clinical practice, could explain the different results. In fact, the post hoc analysis without Yang et al. [17] (Figure 5) showed a low heterogeneity for both subgroups of studies employing plastic stents and metal stents and a lower overall heterogeneity. The latter might be explained in part by the employment of different stent types and, therefore, a different therapeutic protocol with RFA, as previously explained. Furthermore, despite our inclusion of only RCTs, the overall quality of the evidence assessed by GRADE was deemed low. In particular, concerns regarding bias were notable: two of the studies were considered at high risk of bias [16,20], and one study was considered to have some concerns [17]. For that reason, we performed a sensitivity analysis without the studies with a high risk of bias. While the pooled HRs for overall survival were comparable after the sensitivity analysis, the pooled HRs for stent patency did not show a statistical difference between the groups. Excluding two of the four studies originally considered in the analysis may result in a lack of enough power to evaluate statistical significance.

In summary, our findings highlight the nuanced role of RFA in the palliative treatment of CCA. The potential for RFA to enhance OS and stent patency, especially with repeated applications, offers a promising avenue for improving patient outcomes in CCA. Future research should aim to elucidate the cumulative effects of RFA and its optimal integration with stenting techniques, thereby refining treatment paradigms for this challenging malignancy.

## 5. Conclusions

The combination of RFA plus plastic stenting demonstrated a trend toward improved OS in patients with unresectable CCA. Furthermore, stent patency was enhanced in the RFA-treated group. However, based on the current evidence from RCTs, the combination of endoluminal RFA plus stenting for the treatment of unresectable CCA cannot be recommended yet. Further investigation through additional RCTs is warranted to explore the potential cumulative effect of RFA in this population.

## Figures and Tables

**Table 1:** Baseline characteristics of included studies.

Study	Country	Number of Patients	Age ^†^ (years)	Female Sex, *n* (%)	Tumor Location, *n* (%)	Type of Stent	Technique
Yang, 2018 [17]	China	65	62	32 (49)	Bismuth I–II: 19 (29) Distal: 46 (71)	Plastic	Endoscopic
Andrasina, 2021 [20]	Czech Republic	43	66	17 (40)	Bismuth II: 7 (16) IIIa: 15 (35) IIIb: 3 (7) IV: 18 (42)	Metal	Percutaneous
Gao, 2021 [18]	China	147	68 *	85 (49) *	Bismuth I: 18 (12) II: 16 (11) III: 13 (9) Distal: 100 (68)	Plastic	Endoscopic
Kang, 2022 [16]	South Korea	30	72	10 (33)	Bismuth II: 5 (16) IIIa: 8 (27) IIIb: 6 (20) IV: 11 (37)	Plastic	Endoscopic
Jarosova, 2023 [19]	Czech Republic	85	70	33 (39)	Bismuth I: 9 (11) II: 14 (16) III: 28 (33) IV: 22 (26) Distal: 12 (14)	Metal/plastic	Endoscopic

* Data in the entire study population, not just in patients with cholangiocarcinoma; ^†^ mean or median; RFA: radiofrequency ablation.

**Table 2:** Summary of findings and GRADE profile.

Participants (Studies) Follow-Up	Risk of Bias	Inconsistency	Indirectness	Imprecision	Publication Bias	Overall Certainty of Evidence	Relative Effect (95% CI)	Comments
Overall survival								
370 (5 RCTs)	very serious ^a^	not serious	not serious	serious ^b^	none	⨁◯◯◯ Very low	HR 0.62 (0.36 to 1.07)	The evidence about the effect of RFA is very uncertain. The RFA has little to no effect on overall survival, but the evidence is very uncertain.
Patency								
222 (4 RCTs)	very serious ^a^	not serious	not serious	not serious	none	⨁⨁◯◯ Low	HR 0.64 (0.45 to 0.90)	The evidence suggests that RFA results in a slight increase in stent patency.

RCTs: randomized controlled trials; CI: confidence interval; HR: hazard ratio; RFA: radiofrequency ablation. ^a^ Two included studies presented a high risk of bias in two domains (deviation from the intended intervention in the measurement of the outcome and concerning the randomization process) (kang, 2022 [16] and Andrasina, 2021 [20]); ^b^ the low number of included studies and the wide pooled HR led to a downgrade for this domain.

## Data Availability

Given that the meta-analysis utilized data from research already available in the public domain, all study materials and data are publicly accessible. The authors of the meta-analysis do not have access to the patient-level data from the individual studies incorporated. It is recommended that researchers seeking access to individual-level data from the studies included in this meta-analysis directly reach out to the corresponding authors of each specific study for such inquiries.

## Supplementary Materials

The following supporting information can be downloaded at https://www.mdpi.com/article/10.3390/cancers16071372/s1: Table S1: PRISMA checklist.

## Appendix A

(cholangiocarcinoma OR ((bile duct OR biliary) AND (cancer OR tumor OR malignancy)) OR (malignant biliary obstruction OR cholestasis)) AND (“endoluminal radiofrequency” OR “endobiliary radiofrequency” or “endoluminal biliary radiofrequency” or “intraductal radiofrequency” OR “endoscopic radiofrequency”) AND (“stent” OR “stenting” OR “stents” OR “SEMS”).
